# Posttraumatic growth related to the COVID‐19 pandemic among individuals with lived experience of psychiatric disorder

**DOI:** 10.1002/jts.22884

**Published:** 2022-11-02

**Authors:** Catrin Lewis, Katie Lewis, Bethan Edwards, Claudia Evison, Ann John, Holly Pearce, Lawrence Raisanen, Natalie Richards, Alice Roberts, Ian Jones, Jonathan I. Bisson

**Affiliations:** ^1^ National Centre for Mental Health, Division of Psychological Medicine and Clinical Neurosciences Cardiff University School of Medicine Cardiff United Kingdom; ^2^ National Centre for Mental Health, PÂR Cardiff University School of Medicine Cardiff United Kingdom; ^3^ National Centre for Mental Health, Population Data Science Swansea University Medical School Swansea United Kingdom

## Abstract

Although the COVID‐19 pandemic has been shown to be detrimental to mental health, it may hold a parallel potential for positive change. Little is known about posttraumatic growth (PTG) as a potential outcome for individuals with lived experience of psychiatric disorders following trauma exposure, especially in the context of the COVID‐19 pandemic. Participants were 1,424 adults with lived experience of a psychiatric disorder who took part in a longitudinal study of mental health during the COVID‐19 pandemic conducted by the National Centre for Mental Health. PTG was measured using the Posttraumatic Growth Inventory–Short Form (PTGI‐SF). Factors hypothesized to be associated with PTG were investigated using linear regression. The mean participant PTGI score was 12.64 (*SD* = 11.01). On average, participants reported the highest scores on items related to appreciation of life and lowest on those related to spiritual change subscale. We found the strongest evidence of associations between higher levels of PTG and higher scores on assessment items related to perceived social support, *B* = 2.86; perceptions of the pandemic as traumatic, *B* = 4.89; and higher psychological well‐being, *B* = 0.40. Taken together, we did not observe evidence of widespread PTG related to the COVID‐19 pandemic among individuals with lived experiences of psychiatric disorders.

In March 2020, the World Health Organization (WHO) declared the COVID‐19 outbreak to be a global pandemic (Cucinotta & Vanelli, 2020). To save lives and safeguard against health systems becoming overwhelmed, governments around the world enacted unprecedented social distancing and lockdown measures to mitigate the spread of the virus. These measures, combined with the threat to health and life created by the virus itself, created an unparalleled population‐level stressor, with health, social, and economic consequences that will impact the world for decades to come.

Notwithstanding methodological limitations, elevated rates of depression and anxiety have been well established in the general population (Xiong et al., 2020). Similarly, there is evidence of COVID‐19–related posttraumatic stress disorder (PTSD), with prevalence estimates ranging from under 1% to over 60% within study samples (Zhang et al., 2021). Based on the question “During the COVID‐19 crisis, how has your mental health been?,” work we conducted early in the pandemic found that 60% of a sample of participants who had experienced a psychiatric disorder reported their mental health was “worse than usual” or “much worse than usual” (K. Lewis et al., 2022). This was in line with other studies that reported a similar detrimental impact on individuals with a history of psychiatric disorder (O'Connor et al., 2021). An unexpected finding of our work was that 10% of the sample reported mental health that was “better than usual” or “much better than usual.” Despite mounting evidence of maladaptive responses to the pandemic, it is unlikely that negative outcomes are ubiquitous.

Although both positive and negative psychological responses to outbreaks of infectious disease are possible, few studies have considered the potential for positive change, especially in the context of individuals who have experienced a psychiatric disorder. Some research has focused on positive outcomes of previous outbreaks, such as severe acute respiratory syndrome (SARS); studies conducted in the general population reported positive changes, such as self‐empowerment and increased compassion (Chew et al., 2020), during this outbreak. Limited research has focused on positive mental health outcomes arising from the COVID‐19 pandemic.

Posttraumatic growth (PTG) refers to a positive psychological change experienced as a result of the struggle with challenging life events (Calhoun & Tedeschi, 2014). PTG can manifest in many ways, categorized into five broad domains that can arise independently or coexist: appreciation of life, new possibilities, spiritual change, closer relationships, and personal strength (Cann et al., 2010). Initiated by the disruption of core beliefs, Calhoun and Tedeschi (2014) theorized that the process of growth is multifaceted, with both internal factors (e.g., personality traits, cognitive style) and external factors (e.g., social support) playing a part. Traumatic events can create seismic challenges to pretrauma assumptions and views related to the self, others, and the world. Through the process of rebuilding these beliefs, growth can occur (Calhoun & Tedeschi, 2014). PTG is distinct from resilience by virtue of a progression beyond pretrauma levels of psychological functioning. The timing of PTG is uncertain: Whereas some research has indicated that a minimum of 6 months must elapse following trauma exposure for the transformative process that creates PTG to occur, contradictory findings provide evidence that the strongest PTG happens before 6‐months posttrauma (Wu et al., 2019).

PTG development has been well‐documented after exposure to various traumatic events, including life‐threatening illnesses, physical and sexual assault, and military combat. In a meta‐analysis, Wu et al. (2019) reported a pooled prevalence of 53% for moderate‐to‐high PTG across 26 studies of trauma‐exposed participants (Wu et al., 2019). Taken together, meta‐analytic findings indicate associations between PTG and several key variables, including features of the traumatic stressor; demographic characteristics; cognitive processes, such as positive reappraisal; social processes, such as perceived support; peritraumatic distress responses; and positive outcomes, such as higher levels of psychological well‐being (Wu et al., 2019). Whether PTG can arise in response to stressors such as the COVID‐19 pandemic is uncertain. Studies have reported PTG in the general population and among health care workers, but the findings have been inconsistent, and some studies have reported only low rates (Asmundson et al., 2021; Finstad et al., 2021; Hyun et al., 2021). Although the COVID‐19 pandemic has impacted the lives of a high proportion of the global population, levels of exposure have not been uniform. The pandemic creates a risk an individual will directly experience highly traumatic events, such as the life‐threatening symptoms of COVID‐19, or witness others being critically unwell or dying (Bridgland et al., 2021). At the other end of the spectrum, simply living through the pandemic may be perceived as traumatic. News of the virus spreading around the world, overwhelming health care systems and causing millions of deaths, has been said to represent an existential threat of a magnitude that can precipitate outcomes such as PTSD and PTG, but this remains under debate (Shevlin et al., 2020).

Individuals with psychiatric disorders may have been disproportionately affected by the COVID‐19 pandemic due to increased vulnerability to infection and an elevated risk of complications, hospitalization, and mortality (Wang et al., 2021). Possible explanations for these findings include economic deprivation, lifestyle factors, comorbid physical conditions, less compliance with regulations, and a higher likelihood of being exempt from wearing a mask. Studies have also found that psychiatric symptoms such as psychosis, mania, panic attacks, and severe depressive episodes, which may be exacerbated by the pandemic, can also be perceived as traumatic (Lewis et al., 2018). In addition to the potentially heightened risk of exposure to traumatic aspects of the pandemic, there is evidence that people with lived experience of psychiatric disorder may be particularly vulnerable to the development of PTSD, anxiety, and depression following exposure to traumatic life events (Grubaugh et al., 2011). PTG is initiated when cognitive disequilibrium triggered by a traumatic event elicits a process of automatic and intrusive rumination (Calhoun & Tedeschi, 2014). This later gives way to deliberate rumination, which facilitates growth. The process is challenging, and for individuals with an active psychiatric disorder, it may be complicated by preexisting cognitive disequilibrium and symptoms such as intrusive rumination and overvalued ideas. Little is known about PTG development in this population, especially in response to the pandemic.

Similar to most studies on the mental health impact of COVID‐19, our research to date has focused on negative outcomes. We have previously reported on COVID‐19–related depression, anxiety, and PTSD among participants with lived experience of a psychiatric disorder who took part in a longitudinal study of mental health during the COVID‐19 pandemic conducted by the National Centre for Mental Health (NCMH). The finding of improved mental health among a significant minority of participants early in the pandemic provided a rationale to explore PTG within the same cohort of participants. Few prepandemic studies have investigated PTG among individuals with psychiatric disorders, and most of these studies have focused on PTG directly attributable to the trauma of psychopathology, such as psychosis, rather than PTG initiated by external stressors. Knowledge of factors that facilitate PTG has value in informing the response strategy to COVID‐19 and other infectious disease outbreaks in the future. Although associations between PTG and psychological distress variables such as depression, anxiety, and PTSD have been inconsistent, the overall evidence suggests that PTG ameliorates the detrimental impact of trauma (Linley & Joseph, 2004).

In the present study, we aimed to investigate COVID‐19–related PTG within a sample of participants with lived experience of psychiatric disorders (i.e., anxiety, depression, obsessive–compulsive disorder [OCD], bipolar disorder, schizophrenia/psychosis, PTSD, complex PTSD [CPTSD], eating disorders, personality disorders, alcohol or other drug misuse, autism spectrum disorders, and attention deficit hyperactivity disorder [ADHD]). The objectives were to (a) determine the prevalence of PTG and (b) ascertain key factors associated with PTG in this population. Based on previous literature, we hypothesized that COVID‐19–related PTG would be associated with younger age (i.e., an inverse association between age and ratings of PTG; being female), higher income, higher levels of perceived social support, a perception of the pandemic as traumatic, working in a role that entailed virus exposure, testing positive for COVID‐19, having a loved one who tested positive for COVID‐19, higher levels of psychological well‐being, and lower levels of posttraumatic stress symptoms

## METHOD

### Participants and procedure

Data were obtained from a longitudinal study of mental health during the COVID‐19 pandemic conducted by NCMH in partnership with the National Health Service (NHS) across Wales and England. NCMH hosts a cohort of participants with lived experience of a psychiatric disorder who have been recruited on a rolling basis since 2011 using a variety of systematic approaches in primary and secondary health care services, including the identification of potential participants by clinical care teams and screening of clinical notes. Nonsystematic recruitment approaches have included advertisements and engaging third‐sector organizations to promote the research. Upon joining the cohort, participants are asked to provide clinical information, including mental health diagnoses, and demographic data (e.g., gender, age, ethnicity, employment status). Psychiatric diagnoses are determined by asking participants to self‐report diagnoses received by health professionals or those for which they have received treatment.

**FIGURE 1:**
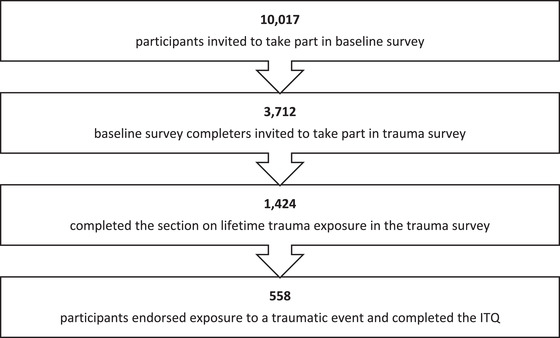
Participant flow. *Note*: ITQ = International Trauma Questionnaire

In June 2020, all members of the NCMH cohort aged 18 years or older who reported lived experience of a psychiatric disorder, had consented to be contacted for future research, and provided an email address (*n* = 10,017, 49.8% of the cohort) were invited to complete an online survey that assessed the impact of the COVID‐19 pandemic on mental health. This baseline COVID‐19 survey included questions on demographic variables (i.e., gender, age, employment, income, living arrangements), mental health and psychiatric service use, and questions related to the COVID‐19 pandemic. In November 2020, a follow‐up survey was sent to the 3,712 participants (*M*_age_ = 44 years, range: 18–94 years) who had completed the baseline survey. This survey repeated many of the baseline questions and asked additional questions specifically related to traumatic stress. Most participants (70.1%) completed the baseline survey in the week of June 15, 2020, with the rest of the sample completing the survey between June 26 and July 30, 2020. Most of the sample (70.29%) completed the follow‐up survey between 5^th^ November and the 11^th^ November 2020, with the rest of the sample completing the survey between 12^th^ November 2020 and 2^nd^ January 2021. See Figure 1 for participant flow.

The mean participant age was 46.70 years (*SD* = 15.41), and 75.35% of the sample (*n* = 1,073) was female. Most participants were White Caucasian (95.3%) and 53.2% were employed, 4.2% of whom were employed as essential workers with exposure to the virus. All participants had self‐reported lived experience of at least one psychiatric disorder, with 28.8% of the sample reporting having received input from specialist mental health services in the 2 weeks before the trauma survey and 69.6% having taken medication related to their mental health diagnosis during that time. Sample characteristics are presented in Table 1.

**TABLE 1:** Sample characteristics

Variable	*M*	*SD*
Age (years)	46.70	15.41
	***n***	**%**
Gender		
Female	1,073	75.3
Transgender female	3	0.2
Male	312	21.9
Transgender male	9	0.6
Gender variant /nonconforming/nonbinary	22	1.5
Ethnicity		
White Caucasian	1,357	95.3
Arab	1	0.1
Asian or Asian British	18	1.3
Black, Black British, Caribbean, or African	1	0.1
Mixed or multiple ethnic groups	17	1.2
Other	15	1.1
Missing	15	1.1
Employment status		
Employed	758	53.2
Retired	230	16.2
Student	117	8.2
Unemployed	317	22.3
Missing	2	0.1
Annual household income (GBP)		
< £10,000	317	22.3
£10,000–£20,000	224	16.2
£20,000–£30,000	225	16.3
> £30,000	497	34.9
Missing	161	11.3
Educational attainment		
None/less than equivalent to GCSE	32	2.3
GCSE or equivalent	248	17.4
A Level or equivalent	281	19.7
University degree or above	768	53.9
Missing	95	6.7
Essential worker with exposure to COVID‐19		
No	1,333	93.6
Yes	60	4.2
Missing	31	2.2
Infection from COVID‐19		
Symptoms of COVID‐19	243	17.1
Tested for COVID‐19	162	11.4
Tested positive for COVID‐19	39	2.7
Mental health diagnoses		
Bipolar disorder	191	13.4
Schizophrenia	132	9.3
Depressive disorder	1,097	77.0
Anxiety	1,243	87.3
Eating disorder	209	14.7
Personality disorder	185	13.0
PTSD	233	16.4
Complex PTSD	80	5.6
Autism spectrum disorder	100	7.0
Worst COVID‐19–related traumatic event		
Infection/suspected infection with COVID‐19	14	2.2
Infection/suspected infection of loved one with COVID‐19	12	2.2
Death of a loved one from COVID	18	3.2
Death of a loved one not from COVID‐19	20	3.6
Being in a high‐risk group for severe infection	12	1.9
Working in a role with exposure to the virus	25	4.5
Generalized worry about the pandemic	172	30.8
Lockdown and social distancing restrictions	119	21.3
Worry about finances/employment	15	2.7
Government response to the pandemic	25	4.5
Behavior of others during the pandemic	25	4.5
Use of face coverings	7	1.3
Exposure to news or media coverage	20	3.6
Changes in access to medical care	21	3.8
Other	20	3.6

*Note*: PTSD = posttraumatic stress disorder.

This study was approved by the Wales Research Ethics Committee.

### Measures

#### Demographic characteristics and psychiatric diagnosis information

Self‐reported age, gender, income, and occupational information, including essential worker status and exposure to the virus, were captured in the baseline COVID‐19 survey. Changes to finances and employment during the pandemic were determined in the follow‐up survey. Mental health diagnoses were collected as part of the baseline survey using the question, “Have you ever been diagnosed with or received treatment for a mental health condition?” If participants responded affirmatively, they were asked, “What mental health or neurodevelopmental diagnosis or condition have you been given or received treatment for?,” with instructions to select from a list of options (see Supplementary Materials) or endorse “other” and provide a free‐text response. Diagnoses were categorized as anxiety, depression, OCD, bipolar disorder, schizophrenia/psychosis, PTSD, CPTSD, eating disorder, personality disorder, alcohol or other drug misuse, autism spectrum disorders, and ADHD.

#### COVID‐19–related information and social support

At both assessment points, participants were asked whether they or anyone close to them had experienced symptoms of COVID‐19 and whether they had tested positive (“yes” or “no”). Participants were asked how socially supported they felt by friends and family in the past 2 weeks, with responses rated on a 5‐point scale ranging from 1 (*very poorly*) to 5 (*very well*).

#### PTG

PTG was measured using the 10‐item Posttraumatic Growth Inventory Short–Form (PTGI‐SF; Cann et al., 2010). Response options were reworded to anchor them to the COVID‐19 pandemic instead of the respondent's index traumatic event. Items were scored on a 6–point Likert scale ranging from 0 (*I did not experience this change as a result of the COVID‐19 crisis*) to 5 (*I experienced this change to a very great degree as a result of the COVID‐19 crisis*). The PTGI‐SF includes two items from each of the five subscales of the original PTGI (i.e., Personal Strength, Appreciation of Life, Closer Relationships, New Possibilities, and Spiritual Change). Total scores can range from 0 to 50, with higher scores indicating higher degrees of PTG. In the current sample, Cronbach's alpha was .89, which is identical to the value derived from the original validation study (Cann et al., 2010).

#### COVID‐19–related trauma exposure

Participants were asked if they found any aspect of the COVID‐19 crisis traumatic (“yes” or “no”). If they answered “yes,” they were prompted to give a free‐text description of their most troubling COVID‐19–related experience. These responses were coded by two researchers against a list of traumatic experiences compiled for this study (Table 1). When participants reported multiple traumatic experiences, the first one mentioned was taken as the most troubling. The same two researchers independently judged whether each reported traumatic event met the PTSD criteria outlined in the *International Classification of Diseases and Disorders* (11th rev.; *ICD‐11*; WHO, 2018) as “exposure to an extremely threatening or horrific event or series of events.” There was good agreement between the two researchers, Cohen's κ = .94. If the researchers were unsure whether the traumatic event met the *ICD‐11* criteria, the event was categorized as not meeting the criteria. When coders disagreed, the event was discussed with a third researcher until a consensus was reached.

#### PTSD symptoms

All participants who endorsed a COVID‐19–related traumatic experience were asked to complete the International Trauma Questionnaire (ITQ; Cloitre et al., 2018) to screen for *ICD‐11* PTSD stemming from this experience. The *ICD‐11* formulation of PTSD includes six symptoms organized into three clusters, compared to the *Diagnostic and Statistical Manual of Mental Disorders* (5th ed.; American Psychiatric Association, 2013), which includes 20 symptoms organized into four clusters. Participants were asked to indicate how much they had been bothered by the six core *ICD‐11* PTSD symptoms in the past month, rating responses on a 5‐point Likert scale ranging from 0 (*not at all*) to 4 (*extremely*). Two symptoms reflect the reexperiencing symptom cluster (i.e., upsetting dreams and flashbacks), two reflect the avoidance cluster (i.e., avoiding internal or external reminders), and two correspond with the cluster related to one's sense of current threat (i.e., being hypervigilant or easily startled). Additional items capture functional impairment associated with these symptoms in three domains. Participants were considered to fulfill the criteria for probable COVID‐19–related *ICD‐11* PTSD if they reported a qualifying traumatic event, rated at least one symptom from each cluster with a score of 2 (*moderately*) or higher, and endorsed at least one of the functional impairment items. Probable ICD‐11 COVID‐19–related PTSD according to the ITQ was considered separately from the diagnoses self‐reported by participants (Table 1). Findings related to trauma exposure, PTSD, and factors associated with posttraumatic stress symptoms have been reported elsewhere (C. Lewis, Lewis, Roberts, Edwards, et al., 2022; C. Lewis, Lewis, Roberts, Evison, et al., 2022). In the present study, Cronbach's alpha was .88.

#### Psychological well‐being

Psychological well‐being was measured at the baseline and follow‐up assessments using the WHO‐5 Well‐Being Index (WHO‐5; Topp et al., 2015). Respondents were asked to rate five statements on a Likert scale ranging from 0 (*at no time*) to 5 (*all of the time*). Total scores can range from 0 to 25, with higher scores indicating higher levels of well‐being. In the present study, Cronbach's alpha was .89.

### Data analysis

All analyses were conducted using Stata (Version 16). Sample characteristics were examined using descriptive statistics. The association between key factors (i.e., age, gender identity, income, perceived social support, perception of the pandemic as traumatic, working in a role with exposure to the virus, testing positive for COVID‐19, a loved one testing positive for COVID‐19, psychological well‐being, and posttraumatic stress symptoms) and PTG were first investigated using univariate linear regressions, with total PTGI‐SF score entered as the dependent variable. We then conducted multivariate analysis using the full sample, which included all predictors and confounders but omitted ITQ score, as the ITQ was only administered to a subsample of participants who endorsed the pandemic as traumatic. We conducted a further multivariate analysis for the subsample of participants who completed the ITQ. The Holm method was used to adjust *p* values to account for multiple testing (Holm, 1979).

## RESULTS

The analytic sample included 1,424 participants who completed questions on PTG as part of the follow‐up survey. Using variables collected at the point of entry into the NCMH cohort with complete or near‐complete data, we found that nonresponse to the COVID‐19–related trauma survey was associated with younger age, male gender, never having been employed, minority ethnicity (i.e., any other than White Caucasian), and having a diagnosis of bipolar disorder or schizophrenia. We did not find evidence of an association between nonresponse and a previous PTSD or CPTSD diagnosis (see Supplementary Materials).

### PTG

The mean total PTGI‐SF score was 12.64 (*SD* = 11.01). Mean scores were highest on items from the PTGI Appreciation of Life subscale, with average scores of 2.05 (*SD* = 1.71) for the item related to changed priorities and 1.75 (*SD* = 1.72) for the item related to appreciation of the value of one's own life. Mean scores were lowest on items from the PTGI Spiritual Change subscale, with average scores of 0.51 (*SD* = 1.24) for the item related to stronger religious faith and 0.91 (*SD* = 1.48) for the item related to having a better understanding of religious matters. Mean item‐level PTGI‐SF scores are shown in Table 2.

**TABLE 2:** Posttraumatic Growth Inventory–Short Form (PTGI‐SF) mean item scores

Variable	PTGI domain	*M*	*SD*
Changed priorities about what is important in life	Appreciation of life	2.05	1.71
Greater appreciation for value of own life	Appreciation of life	1.75	1.72
Able to do better things with life	New possibilities	1.19	1.43
Better understanding of spiritual matters	Spiritual change	0.91	1.48
Greater sense of closeness with others	Closer relationships	1.31	1.56
Established a new path for life	New possibilities	0.91	1.46
Know better that can handle difficulties	Personal strength	1.24	1.47
Stronger religious faith	Spiritual change	0.51	1.24
Stronger than thought	Personal strength	1.28	1.58
Learned a great deal about how wonderful people are	Closer relationships	1.49	1.62
PTGI‐SF total score		12.64	11.01

### COVID‐19–related trauma exposure and probable PTSD

A total of 558 participants (39.2%) participants found some aspect of the COVID‐19 pandemic traumatic and, thus, were asked to complete the ITQ. The most frequently reported traumatic experiences were generalized worry about the pandemic (30.8%, *n* = 172) and lockdown or social distancing restrictions (21.3%, *n* = 119). In the total sample, 5.4% (*n* = 77) of participants reported a traumatic experience that was judged to meet the *ICD‐11* criteria for PTSD, and 0.84% of participants (*n* = 13) met the criteria for probable COVID‐19–related PTSD. A more detailed account of the findings related to PTSD is reported elsewhere (C. Lewis, Lewis, Roberts, Evison, et al., 2022).

### Psychological well‐being

For the full sample, mean WHO‐5 scores were 8.66 (*SD* = 5.42) at baseline and 8.04 (*SD* = 5.33) at follow‐up. Among participants who endorsed perceiving some aspect of the pandemic as being traumatic (*n* = 558), mean WHO‐5 scores were 7.72 (*SD* = 5.02) at baseline survey and 7.00 (*SD* = 4.96) at follow‐up.

### Associations between PTGI‐SF scores and key variables

Complete case analyses were conducted. Preliminary analyses indicated no violations of the assumptions of normality, linearity, homoscedasticity, or multicollinearity. After adjusting for potential confounders, other predictor variables, and multiple testing, there was evidence that higher PTGI‐SF scores were most strongly associated with increased perceived social support, *B* = 2.86, 95% CI [1.53, 4.19], *p* < .001; a perception of the pandemic as being traumatic, *B* = 4.89, 95% CI [3.57, 6.21], *p* < .001; and higher levels of psychological well‐being (i.e., WHO‐5 score), *B* = 0.40, 95% CI [0.27, 5.27, *p* < .001. The model as a whole explained 10.9% of the variance. For the subsample of participants who perceived the pandemic as traumatic and completed the ITQ, we found evidence that higher PTGI‐SF scores were most strongly associated with higher levels of psychological well‐being, *B* = 0.67, 95% CI [0.42, 0.93], *p* < .001, and more severe PTSD symptoms (i.e., higher ITQ scores), *B* = 0.35, 95% CI [0.16, 0.53], *p* < .001. The model as a whole explained 10.1% of the variance; These results are presented in table 3 and a correlation matrix in table 4 and 5.

**TABLE 3:** Results of regression analyses

	Unadjusted univariable analyses				Multivariable analysis for the full sample^b^			Multivariable analysis for the subgroup who completed the ITQ^c^		
Variable^a^	*B*	95% CI	*F* (*df*, *df*)	*p*	*B*	95% CI	*p*	*B*	95% CI	*p*
Age^d^	−0.05	[−0.09, −0.01]	6.51(1, 1,414)	.011	−0.06	[−1.11, 0.02]	.004^e^	−0.05	[−1.13, 0.02]	.174
Gender	2.37	[0.98, 3.73]	11.34 (1, 1,395)	.001	1.67	[0.21, 3.13]	.025	1.29	[−1.35, 3.93]	.338
Income	−0.63	[−1.86, 0.60]	1.00 (1, 1,261)	.316	0.73	[−0.56, 2.03]	.286	−0.56	[−2.81, 1.69]	.626
Social support	4.16	[3.03, 5.28]	52.44 (1, 1,226)	< .001	2.86	[1.53, 4.19]	< .001^e^	2.42	[0.13, 4.70]	.038
Pandemic perceived as traumatic	4.00	[2.85, 5.16]	46.26 (1, 1,421)	< .001	4.89	[3.57, 6.21]	< .001^e^			
Work role with exposure to the virus	1.59	[−1.26, 4.44]	1.20 (1, 1,391)	.274	−0.55	[−3.88, 2.78]	.745	−1.55	[−6.29, 3.19]	.520
Tested positive for COVID‐19	3.88	[−0.39, 7.38]	4.74 (1, 1,418)	.030	−0.25	[−4.46, 3.55]	.907	0.26	[−6.07, 6.59]	.935
Loved one tested positive	3.20	[1.40, 5.01]	12.16 (1, 1,320	.001	1.61	[−0.32, 3.55]	.102	2.24	[−0.85, 5.33]	.155
WHO‐5 score	0.19	[0.08, 0.29]	11.80(1, 1,356)	.001	0.40	[0.27, 5.27]	< .001^e^	0.67	[0.42, 0.93]	< .001^e^
ITQ score	0.11	[−0.05, 0.26]	1.82 (1, 542)	.178				0.35	[0.16, 0.53]	< .001^e^

*Note*: WHO‐5 = World Health Organization–Five Well‐Being Index; ITQ – International Trauma Questionnaire; *df* = degree of freedom.

^a^ Gender: 0 = male, 1 = female; income: 1 = < £20,000, 0 = income ≥ £20,000; social support: 1 = socially supported by friends well/very well, 0 = very poorly/poorly/neither poorly nor well; pandemic perceived as traumatic: 1 = yes, 0 = no; work role with exposure to the virus: 1 = yes, 0 = no; tested positive for COVID‐19: 1 = yes, 0 = no; loved one tested positive for COVID‐19: 1 = yes, 0 = no.

^b^ *F*(9, 1,086) = 14.78.

^c^ *F*(9, 395) = 6.02.

^d^ Continuous variable.

^e^ Variable survived adjustment for multiple testing.

**TABLE 4:** Correlation matrix for the full sample

Variable	1	2	3	4	5	6	7	8	9	10
1. PTGI‐SF	–	−.0636*	.0901**	−.0183	.192***	.163***	−.000214	.0364	.0826**	.100***
2. Age		–	−.154***	.0944**	−.0833**	.0574	−.0682*	−.0953**	−.103***	.139***
3. Gender			–	−.0534	.0814**	.0720*	.0343	.0323	.0140	−.0474
4. Income				–	−.114***	.0509	−.0872**	−.0185	−.0504	−.201***
5. Social support					–	−.0608*	−.0389	.0357	.0856**	.279***
6. Pandemic perceived as traumatic						–	.0680*	.0439	.0659*	−.141***
7. Work in role with exposure to virus							–	.100***	.0949**	−.0296
8. Tested positive for COVID‐19								–	.134***	.00417
9. Loved one tested positive									–	−.0337
10.WHO‐5 score										–

*Note*: International Trauma Questionnaire (ITQ) score is absent because it was only administered to participants who perceived the pandemic as traumatic. PTGI‐SF = Posttraumatic Growth Inventory–Short Form; WHO‐5 = World Health Organization–Five Well‐Being Index.

* *p* < .05. **p < .01. ***p < .001.

* *p*1

**TABLE 5:** Correlation matrix for participants who perceived the pandemic as traumatic and completed the International Trauma Questionnaire (ITQ)

Variable	1	2	3	4	5	6	7	8	9	10
1. PTGI‐SF	–	−.08	.06	−.06	.19***	.06	−.00	.02	.11*	.25***
2. Age		–	−.11*	.13**	−.10*	−.11*	−.16**	−.12*	−.10*	.09
3. Gender			–	−.03	.11*	.03	−.01	.02	−.08	−.02
4. Income				–	−.06	.23***	−.13**	−.07	−.14**	−.21***
5. Social support					–	−.19***	.02	.07	.06	.34***
6. ITQ score						–	−.03	−.02	.02	−.42***
7. Work in role with exposure to virus							–	.15**	.20***	.02
8. Tested positive for COVID‐19								–	.09	.00
9. Loved one tested positive									–	.10*
10.WHO‐5 score										–

*Note*: “Pandemic perceived as traumatic” omitted because the ITQ was only administered to participants who endorsed perceiving the pandemic as traumatic. PTGI‐SF = Posttraumatic Growth Inventory–Short Form; WHO‐5 = World Health Organization–Five Well‐Being Index.

* *p* < .05. **p < .01. ***p < .001.

## DISCUSSION

We investigated COVID‐19–related PTG within a large sample of participants with lived experience of psychiatric disorder and found only low levels (i.e., a mean PTGI–SF score of 12.64) of PTG in the sample. Mean PTGI‐SF item scores were highest for items from the PTGI‐SF Appreciation of Life subscale and lowest for those from the Spiritual Change subscale. Although traumatic experiences related to death or near‐death are generally found to increase spiritual change, few study participants lost loved ones to COVID‐19, and the study was conducted in the United Kingdom, where a relatively small proportion of the population describe themselves as religious or spiritual (King et al., 2013).

There was less evidence of PTG in the present study than reported across studies of many other traumatic stressors, including cancer, natural disasters, accidents, and sexual assault (Wu et al., 2019). Findings of COVID‐19–related PTG have been inconsistent, and the present study adds to a growing body of literature that has failed to find evidence of growth, with a pattern of findings similar to those found in the general population (Menculini et al., 2021). Our findings related to low levels of PTG in a sample of participants with psychiatric disorders are consistent with previous findings indicating that individuals with psychiatric disorders may have been less impacted by the pandemic than feared (Pinkham et al., 2020), perhaps due to prior experience of dealing with adversity and social isolation. However, it is also worth noting that the NCMH cohort includes participants with both current and past psychiatric disorders, and it is possible that those who responded were more likely to be well than those who did not. In general, little is known about PTG in the context of individuals with preexisting psychiatric disorders. The process is cognitively challenging, and it is possible that people with disorders characterized by ruminative thinking, such as depression, anxiety, and OCD, struggle to pass from intrusive to deliberate rumination. It is also possible that these individuals have difficulties expressing themselves or have limited social support due to their condition or the perceived stigma associated with it, which may also impact PTG.

The reasons some individuals exposed to traumatic stressors respond with traumatic growth are complex and multifactorial. PTG is said to occur as a result of highly challenging life events that disrupt pretrauma assumptions and views related to oneself, others, and the world (Asmundson et al., 2021; Calhoun & Tedeschi, 2014). It is debatable whether the COVID‐19 pandemic represents such an event for most people. A possible explanation for the present findings is that the COVID‐19 pandemic was not widely experienced as an event that was sufficiently traumatic to precipitate PTG. The COVID‐19 pandemic has been a major life event for most people living in affected countries; however, it may only have been a traumatic event in the true sense of the word to a small proportion of individuals, and only that small proportion includes those likely to respond with PTSD or PTG. In support of this argument, we found evidence of an association between experiencing the pandemic as traumatic and higher ratings of PTG, and within the subsample of participants who perceived the pandemic as traumatic, PTG was associated with higher PTSD scores.

Studies such as the present study, which determined the nature of COVID‐19–related exposures, have observed a small number of participants likely to meet the *ICD‐11* criteria for a PTSD‐qualifying event (Bridgland et al., 2021). The more prevalent experiences reported were related to generalized worry about the virus and its consequences, the disruption created by lockdowns, and concerns regarding finances and employment. Experiences of this nature may be more likely to result in symptoms of anxiety and depression or to exacerbate existing psychiatric symptoms rather than initiate responses such as PTG or PTSD. Simply living through the pandemic may not be sufficiently traumatic to initiate the cognitive processing required for PTG, but it may heighten the risk of exposure to events that are powerful enough to initiate growth. This is consistent with evidence that PTG is associated with more severe trauma exposure or a heavier trauma burden (Schubert et al., 2016). On the contrary, after controlling for confounders and other predictors, we did not find evidence of an association between PTG and personally contracting or losing a loved one to COVID‐19. Similarly, we did not find an association between PTG and working in a role that entailed exposure to the virus, which contrasts with some studies that have reported high levels of PTG among health care workers (Finstad et al., 2021) and prepandemic meta‐analytic findings of an association between PTG and being employed in a role that carries a high likelihood of trauma exposure (Wu et al., 2019). However, the small number of participants in the present study who endorsed these exposures may have resulted in insufficient statistical power to detect an association. In addition, we did not find an association between PTG and symptoms of traumatic stress, although it is worth noting that few participants met the diagnostic criteria for COVID‐19–related PTSD. In contrast, Ikizer et al. (2021) observed a positive correlation between PTG and traumatic stress symptoms assessed during the pandemic among a general population sample in Turkey.

In keeping with previous research (Wu et al., 2019), we found that female gender was associated with higher levels of PTG in univariable analyses, but this was no longer evident after controlling for other predictors and confounders and correcting for multiple testing. We also found evidence of an association between PTG and higher levels of mental well‐being, which underscores the idea that PTG is a positive outcome rather than simply a lack of distress (Morris & Shakespeare‐Finch, 2011). An alternative explanation for this finding is that the instruments used to measure PTG and well‐being assess similar concepts that are likely to be correlated. In addition, we found evidence of an association between PTG and perceived social support, which is in line with prepandemic and COVID‐19–related research (Northfield & Johnston, 2021). Combined with previous findings of the inverse association between social support and trauma‐related symptoms whereby social support buffers against the development of PTSD (Brewin et al., 2000; Prati & Pietrantoni, 2009), this finding indicates an important protective role of social support during the pandemic. This is noteworthy given that measures taken to protect physical health have often diminished social support. The specific mechanisms by which social support and PTG are associated remain uncertain, and the association may be bidirectional; that is, PTG may facilitate adjustment to a traumatic event by acting as a predictor of growth. Conversely, the perception of positive change associated with PTG may result in the strengthening of current relationships and the creation of new connections, which may, in turn, generate social support as an outcome. Both prepandemic and COVID‐19–related research has found evidence of an association between PTG and younger age (Northfield & Johnston, 2021; Wu et al., 2019), which may be attributable to younger people being more flexible in their ability to change their views. The present results did not replicate this finding. Although some researchers have found an association between PTG and higher income (Yıldız, 2021), which may be due to financial resources buffering against stressful experiences and facilitating growth, we did not find evidence of such an association. There are no clear explanations for these nonsignificant findings, but the low levels of PTG within the sample may have resulted in a lack of association with these factors.

To our knowledge, this was the first study to explore PTG related to the COVID‐19 pandemic among participants with lived experience of psychiatric disorders. The sample was large and included participants with a range of diagnoses. We anchored the measurement of PTG to the pandemic to give an estimate of COVID‐19–related growth. We also determined the specific COVID‐19–related traumatic events that troubled participants the most and measured trauma‐related symptoms anchored to these events using the ITQ, which represents the gold standard self‐report measure of *ICD‐11* PTSD.

There were several limitations that should be acknowledged. First, due to restrictions posed by the pandemic, participants completed an online survey, and only participants with an email address were invited to take part. This may have underrepresented older participants and those from very low‐income households. In addition, the wide‐ranging recruitment strategies used to build the cohort, response biases associated with the survey, and overrepresentation of female participants limit the generalizability of the results. For cases in which participants listed more than one “most troubling” trauma exposure, the one listed first was categorized as the index event. Although this approach had clear limitations, it rarely occurred (*n* = 79, 5.5%), and it never resulted in the omission of a PTSD‐qualifying event. In some cases, reported traumatic experiences fell into multiple categories. Two researchers independently assigned them to the most relevant group, and there was a high degree of agreement. The survey was administered 7.5 months after the first lockdown in the United Kingdom at a point when many of the participants in England and Wales were in a national lockdown and before vaccines were available, meaning that PTG was measured during an ongoing situation. The timeframe for the development of PTG is uncertain (Wu et al., 2019), but some researchers have found that the process of growth may occur in the context of ongoing distress (Shakespeare‐Finch & Lurie‐Beck, 2014) Thus, the results indicate the magnitude of PTG during the pandemic, which can be further examined at follow‐up assessments. Finally, the range of adverse outcomes that can occur posttrauma along with PTSD, including acute stress and moral injury, neither of which we assessed, must be acknowledged.

The COVID‐19 pandemic is an unprecedented population‐level stressor, and research findings related to its impact on mental health should inform the response strategy to future events of a similar nature. There is a clear need to consider the psychological impact of measures taken to protect physical health and ensure that these are not harmful to mental health. Given the potentially beneficial influence of PTG, it is logical to work toward maximizing this process in the aftermath of trauma. For example, social support is commonly found to be associated with PTG, and there is a potential for peer support initiatives to enhance PTG and ameliorate the negative mental health consequences of stressors such as the COVID‐19 pandemic.

The present findings should be replicated in additional samples of participants with and without lived experience of a psychiatric disorder. Future follow‐up assessments of this cohort are required to determine the ongoing trajectory of change and whether PTG is associated with other outcomes in the future. This is particularly important, as the pandemic has progressed through many stages, and with new variants emerging, it is yet to end. A lack of certainty regarding timeframes for the development of PTG also drives a need for longitudinal research. This may include participant follow‐ups that utilize routinely collected health‐service data to minimize the bias associated with self‐report data. Concurrent qualitative research would serve a useful function in determining the factors that underlie COVID‐19–related PTG or lack thereof. More generally, the nature and implications of PTG have been debated and there remains a need for a better empirical understanding of the construct (Infurna & Jayawickreme, 2019). Whereas PTG was originally conceptualized as reflective of genuine growth resulting from a struggle, some scholars have argued that PTG reflects a maladaptive response that stems from avoidance or dissociation rather than a truly positive outcome (Hobfoll et al., 2007). Although the present finding of an association between PTG and psychological well‐being suggests otherwise, additional work is needed to explore this further. There has been a lack of truly longitudinal research related to PTG, and whether retrospective reports of growth captured by instruments such as the PTGI‐SF reflect true change is unclear. The available measures rely on participants accurately recalling an earlier psychological state, which is cognitively challenging and often unreliable (Jayawickreme et al., 2021). Driven by adaptive processes such as self‐empowerment, participants may be motivated to perceive growth after a traumatic life event, even in its absence (Jayawickreme et al., 2021). Suggestions to improve the validity of PTG measurements include capturing both state and trait measures of psychological functioning, complementing self‐report measures of PTG with data provided by other informants, and incorporating behavioral measures (Davis et al., 2021). There may be additional benefits in personalizing measures of PTG to specific traumatic events. For example, the nature of PTG may be different in the context of a pandemic compared to a terrorist attack, and tailored instruments may enable more precise measurement.

## OPEN PRACTICES STATEMENT

The study reported in this article was not formally preregistered. Neither the data nor the materials have been made available on a permanent third‐party archive; requests for the data or materials can be sent via email to the lead author at LewisCE7@Cardiff.ac.uk.

## AUTHOR NOTE

The authors would like to thank all participants for kindly giving their time to take part in this research.

## Supporting information


**Table S1**: Predictors of non‐response to COVID‐19 trauma survey
