# Supplementary material for: Posttraumatic growth related to the COVID‐19 pandemic among individuals with lived experience of psychiatric disorder
Source: J Trauma Stress. 2022 Nov 2;35(6):1756–68. doi: 10.1002/jts.22884 (PMC9877956; doi:10.1002/jts.22884)
Supplement: Supplementary file 1 — Table S1: Predictors of non‐response to COVID‐19 trauma survey [file JTS-35-1756-s001.docx]

**Supplementary material**

**Table S1: Predictors of non-response to COVID-19 trauma survey**

*Results of regression analyses (univariate)*

| Variable | OR | | 95% CI | | P |
| --- | --- | --- | --- | --- | --- |
| Age | 0.98 |  | | 0.98-0.99 | 0.000 |
| Gender | 0.85 |  | | 0.74-0.97 | 0.000 |
| Ever employed | 0.73 |  | | 0.64-0.82 | 0.000 |
| Minority ethnicity | 1.56 |  | | 1.15-2.10 | 0.004 |
| Diagnosis of bipolar disorder | 1.24 |  | | 1.05-1.46 | 0.013 |
| Diagnosis of schizophrenia | 1.25 |  | | 0.99 -1.55 | 0.050 |
| Diagnosis of PTSD/CPTSD | 0.89 |  | | 0.76-1.05 | 0.173 |

Age – continuous; gender coded as 0 = male, 1 = female; ever employed coded as 1 = yes, 0 = no; minority ethnicity coded as 1 = yes, 0 = no; diagnosis of bipolar disorder coded as 1 = yes, 0 = no; diagnosis of schizophrenia coded as 1 = yes, 0 = no; diagnosis of PTSD/CPTSD coded as 1 = yes, 0 = no.

**Supplementary material**

**Mental health diagnoses that could be endorsed by participants in the baseline survey in response to the following questions:**

**Have you ever been diagnosed with or received treatment for a mental health condition?** [Yes, No, Not sure, Prefer not to say]

IF YES:

**What mental health conditions have you been diagnosed with or received treatment for?** (SELECT ALL THAT APPLY)

*Mood Disorders*

Depressive disorder (Depression, Major Depression)

Bipolar disorder (Manic depression)

Mania/Hypomania

Premenstrual dysphoric disorder (PMDD) / Premenstrual syndrome (PMS)

*Psychotic Disorders*

Schizophrenia

Schizoaffective disorder

Psychosis

*Anxiety Disorders*

Anxiety (Generalised Anxiety Disorder, GAD)

Agoraphobia

Panic disorder

Phobias

Obsessive Compulsive Disorder (OCD)

*Autistic Spectrum Disorders*

Autism

Asperger’s syndrome

*Eating Disorders*

Anorexia

Bulimia

Binge Eating Disorder

*Attention Deficit Hyperactivity Disorder (ADHD)*

*Post-Traumatic Stress Disorder (PTSD)*

*Complex Post-Traumatic Stress disorder (CPTSD)*

*Personality Disorders*

Borderline personality disorder (emotionally unstable personality disorder)

Other Personality Disorder

*Alcohol and drug problems*

Alcohol dependence / misuse

Dependence / misuse of other drugs

*Dementia*

*Perinatal/Postpartum disorders*

Mood disorder in pregnancy

Postpartum psychosis (Postnatal psychosis / Puerperal Psychosis)

Postnatal depression (Postpartum Depression)
